# Monitoring conformational changes in the human neurotransmitter transporter homologue LeuT with ^19^F‐NMR spectroscopy

**DOI:** 10.1111/jnc.16278

**Published:** 2024-12-16

**Authors:** Alberto Daminato, Claus J. Loland, Eurico J. Cabrita

**Affiliations:** ^1^ Associate Laboratory i4HB—Institute for Health and Bioeconomy, NOVA School of Science and Technology Universidade NOVA de Lisboa Caparica Portugal; ^2^ UCIBIO, Department of Chemistry, NOVA School of Science and Technology Universidade NOVA de Lisboa Caparica Portugal; ^3^ Laboratory for Membrane Protein Dynamics, Department of Neuroscience, Faculty of Health and Medical Sciences University of Copenhagen Copenhagen Denmark

**Keywords:** ^19^F‐NMR (fluorine nuclear magnetic resonance), conformational dynamics, ion coupling, LeuT (Leucine Transporter), neurotransmitter transporters, NSS (Neurotransmitter:sodium symporters)

## Abstract

Neurotransmitter:sodium symporters (NSS) reuptake neurotransmitter molecules from the synaptic space through Na^+^‐coupled transport. They are thought to work via the alternating access mechanism, exploring multiple configurations dictated by the binding of substrates and ions. Much of the current knowledge about these transporters has been derived from examining the structure of the Leucine Transporter (LeuT), a bacterial counterpart to human NSSs. Multiple crystal structures of LeuT provided valuable information regarding the steps involved in this mechanism. Dynamical data connecting the crystal structure to the transport cycle are critical to understanding how ligands are translated through the membrane. In the present study, we applied ^19^F‐based nuclear magnetic resonance (NMR) spectroscopy to ^19^F labelled LeuT to monitor how substrates and ions binding affect the conformations of the transporter. By selecting mutations and ligands known to affect the conformational equilibrium of LeuT, we identified and assigned four NMR resonances to specific conformational states of LeuT. We observe that Na^+^ ions produce closure of the extracellular vestibule to a state similarly induced by Na^+^ and substrates. Conversely, K^+^ ions seem to shift the conformational equilibrium toward inward‐facing intermediates, arguably by competing with Na^+^. The present study assembles a framework for NMR‐based dynamical studies of NSS transporters and demonstrates its feasibility for tackling large membrane LeuT‐fold transporters.

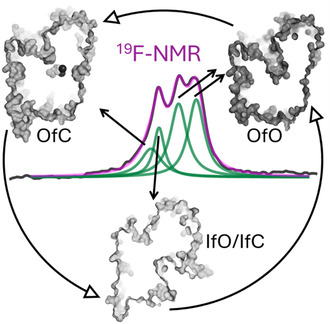

## Graphical abstract

Neurotransmitter:sodium symporters (NSS) reuptake neurotransmitter molecules from the synaptic space through Na^+^‐coupled transport. Much of the current knowledge about these transporters and the multiple configurations dictated by the binding of substrates and ions, has been derived from examining the structure of the Leucine Transporter (LeuT), a bacterial counterpart to human NSSs. By applying ^19^F‐based nuclear magnetic resonance (NMR) spectroscopy to ^19^F labelled LeuT, we observed that Na^+^ ions produce closure of the extracellular vestibule to a state similarly induced by Na^+^ and substrates. Conversely, K^+^ ions seem to shift the conformational equilibrium toward inward‐facing intermediates, arguably by competing with Na^+^.

**Abbreviations**
ADHD: attention‐deficit hyperactivity disorder
BGT1: betaine GABA Transporter 1
BTFA: 3‐bromo‐1,1,1‐trifluoropropanone
DAT: Dopamine Transporter
DDM: n‐dodecyl‐b‐D‐maltoside
EL: extracellular loop
EPR: electron paramagnetic resonance
FRET: Förster resonance energy transfer
GAT: GABA transporter
HDX‐MS: Hydrogen/Deuterium eXchange Mass Spectrometry
IfC: inward facing closed
IfO: inward facing open
IPTG: isopropyl b‐D‐1‐thiogalactopyranoside
LeuT: Leucine Transporter
MDMA: 3,4‐methyl enedioxy methamphetamine
NET: norepinephrine transporter
NMR: nuclear magnetic resonance
NSS: neurotransmitter:sodium symporters
OD: optical density
OfC: outward facing closed
OfO: outward facing open
SERT: serotonin transporter
SPA: scintillation proximity assay
TCEP: tris‐(2‐carboxyethyl)‐phosphine
TM: transmembrane helix

## 1 INTRODUCTION

Neurotransmission is a finely tuned mechanism. It happens at the level of the synapses, interneuronal crossroads where electrical stimuli are converted into chemical messages in the form of neurotransmitters (Jessell & Kandel, 1993). Action potential travelling down the presynaptic neuron causes the release of these small molecules into the synaptic cleft, where they can interact and activate receptors located on the membrane of post‐synaptic neurons. Signal transmission is then actively terminated by integral membrane transporters located on the surface of presynaptic neurons and glial cells (Amara & Kuhar, 1993). These neurotransmitter:sodium symporters (NSS) harness sodium and chloride electrochemical gradients to drive the otherwise energetically unfavourable movement of neurotransmitters across the membrane (Rudnick, 1998). Mammalian members of this family include transporters for biogenic amines, such as dopamine (DAT), norepinephrine (NET) and serotonin (SERT), amino acids, such as γ‐aminobutyric acid (GAT‐1‐4), glycine (GlyT‐1‐2), and osmolytes as betaine (BGT1) and creatine (CT1). The importance of this class of transporters is highlighted by the intense behavioural effect of drugs (e.g. cocaine, amphetamines and MDMA) and medicines (e.g. anti‐depressants, anti‐epileptics and ADHD medications) that interact with their function. Indeed, malfunctions of NSS transporters contribute to several pathophysiological conditions, such as anxiety, depression, epilepsy, ADHD and schizophrenia (Kristensen et al., 2011; Shannon et al., 2000). Despite their diversity in substrate specificity, these transporters share a common architecture consisting of 12 transmembrane domains (TM) and the transport of (at least) one Na^+^ and one Cl^−^ ion together with their specific substrate per transport cycle. The transport process is believed to follow the alternating access mechanism, postulated almost 60 years ago (Mitchell, 1957; Oleg, 1966; Vidaver, 1966), allowing the transporters to couple conformational changes with substrate binding/release events by alternatively exposing the binding site(s) to the extracellular or intracellular environment. Much of what is today known about these transporters has been inferred by looking at the structure of a bacterial homologue of the human NSSs, the Leucine Transporter (LeuT) from the thermophile bacterium *Aquifex aeolicus* (Deckert et al., 1998; del Alamo et al., 2022; Loland, 2015; Penmatsa & Gouaux, 2014). This amino acid transporter was first crystallised by Yamashita et al. (2005) nearly 20 years ago, and it revealed a protein organised in 12 TMs, where the first TMs 1–5 shared a similar yet inverted (in the membrane plane) structure with the following *TMs 6–10*, and the last two TMs arranged in a dimerization domain. Two sodium ions, bound at sites Na1 and Na2, form hydrogen bonds with the amino acid substrates and, together with the unwound region of TM 1 and TM 6, mould the central binding site (S1). This structural organisation has been referred to as the LeuT‐fold and, since its first observation, has been identified in several membrane transporters belonging to the most distant‐related organisms (for a recent review of the LeuT fold see ref. (del Alamo et al., 2022)). Given its ubiquitous appearance, and striking similarity with the human NSS transporters (Singh, 2008), substantial efforts have been dedicated to investigating LeuT‐fold structural and dynamical features. Building on a plethora of tridimensional structures (more than 50), EPR, FRET, HDX‐MS, cysteine accessibility and computational methods bridged the gaps between the crystallographic structures and created a comprehensive model of LeuT‐mediated transport mechanism. Interestingly, despite being regarded as one of the best methods to investigate protein dynamics (Henzler‐Wildman & Kern, 2007), nuclear magnetic resonance (NMR) is virtually absent from the toolkit of techniques that have been applied to investigate LeuT and, more broadly, NSS transporters. This can mainly be attributed to (i) the increased spectral complexity because of the large number of overlapping signals and (ii) sensitivity losses because of the rapid relaxation of the NMR signals of the large, slowly tumbling transporters (Claridge, 2016). As a consequence, traditional NMR methods are usually limited to proteins with masses up to 30 kDa or less (Wagner, 1993). However, advances in transverse‐relaxation‐optimised spectroscopy (TROSY) (Pervushin et al., 1997), ultra‐high‐field magnets (Callon et al., 2021) and labelling techniques, including deuteration and methyl group isotopic labelling (Gans et al., 2010; Kainosho et al., 2006; Mund et al., 2013), have pushed the size close to 1 MDa. In this respect, the use of spectroscopic probes, such as ^19^F‐NMR, has also emerged as an important tool for studying large protein systems (for recent reviews see Gronenborn, 2022; Marsh & Suzuki, 2014; Picard & Prosser, 2021). This is particularly because of ^19^F high sensitivity, large chemical shift dispersion (Gerig, 1989) and absence of background signals (Harper et al., 2003).

**FIGURE 1:**
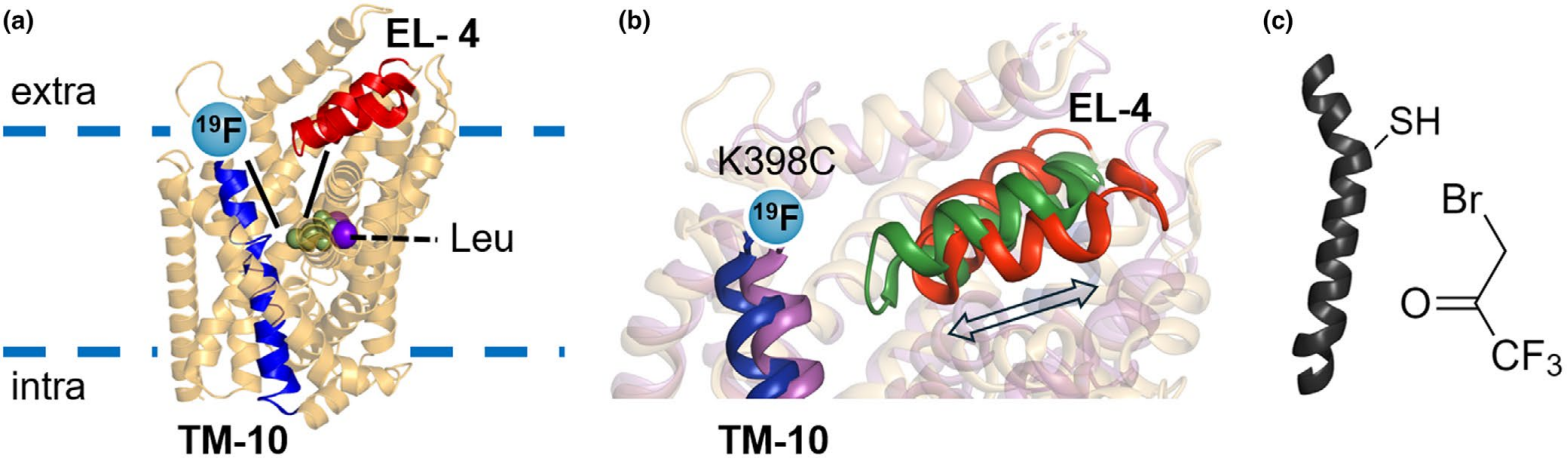
LeuT labelling and probe positioning. (a) Cartoon representation of LeuT in the outward closed conformation (PDB‐ID 2A65) parallel to the plane of the membrane with TM10 (blue) and EL‐4 (red) highlighted. Leucine (green) and Na^+^ (purple) are represented as spheres in the central binding site (S1). The light blue circle indicates the approximate position of the ^19^F BTFA label at the extracellular end of TM10. The smaller blue lines indicate the approximate location of the binding vestibule. (b) Zoomed in view of the superimposed extracellular side of outward closed LeuT (PDB‐ID 6XWM) and outward open LeuT (PDB‐ID 3TT1) displaying the large movements of EL‐4 following the closure of the extracellular gate. EL‐4 is depicted red for outward open LeuT and green for outward closed LeuT. The double‐sided arrow indicates that EL‐4 undergoes a rocking movement regulating the opening/closure of the extracellular gate. (c) Cartoon depiction of a cysteine residue in an α‐helix (left) and structure of the ^19^F probe BTFA (right).

In this work, we have followed the ^19^F labelling strategy to explore its application to elucidate critical dynamic processes of the LeuT transport cycle. For this purpose, we have labelled a mutant cysteine (K398C) located at the extracellular side of LeuT (TM10) with the fluorinated probe BTFA and used it to record (1D) ^19^F‐NMR spectra (Figure 1a). Four ^19^F‐NMR peaks were identified and, by combining knowledge from previous studies, they were systematically assigned to specific states of the LeuT transport cycle. Equipped with this ‘conformational framework’, we then observed the effect of sodium ion binding on the extracellular side of LeuT and obtained preliminary results on the effect of K^+^ binding. Overall, we demonstrate the feasibility of investigating the conformational landscape of LeuT using ^19^F‐NMR and open an avenue for future NMR investigation of the human NSS transporters.

## 2 MATERIALS AND METHODS

The authors state that no blinding procedures were performed, and institutional ethics approval was not required.

### 2.1 Expression and Purification of LeuT

*LeuT encoding DNA from A. aeolicus*, fused to a thrombin protease cleavage site and an octahistidine tag at the C‐terminus, was cloned into a pET16b vector. Mutations were inserted into a LeuT construct with the pre‐existing mutation K398C. Full gene sequences were verified by DNA sequencing (Eurofins Genomics and STAB VIDA).

C41‐strain Escherichia coli cells (from C.Loland laboratory) were transformed with the desired LeuT construct and cultivated in Lysogeny Broth added with 100 mg/mL^−1^ ampicillin at 37°C and shaking. When OD (Optical Density) reached 0.6, 100 mM isopropyl b‐D‐1‐thiogalactopyranoside (IPTG, cat.no. IPTG‐RO, Sigma) was added to induce expression, before incubation at 18°C and shaking for 22 h. Cells were collected by centrifugation at 3700 G and subsequently broken by a triple passage through a basic Z Cell Disruptor (constant system) at 21 kPsi and 4°C. The membrane fraction was isolated by ultracentrifugation at 120 000 g (Beckmann Coulter) for 1.5 h. Isolated membranes were resuspended in 50 mM Tris–HCl (pH 8), 200 mM KCl, 0.5 mM tris‐(2‐carboxyethyl)‐phosphine (TCEP, cat.no. 646547, Sigma), 20% glycerol (buffer A) using a glass homogeniser and 1% (w/v) of n‐dodecyl‐b‐D‐maltoside (DDM, cat.no. D310, Anatrace) was added to extract and solubilise LeuT before incubation at 4°C for 2 h. LeuT was immobilised on HisPur Cobalt Resin (cat.no. 899648, Thermo Fisher) and washed with Buffer B (20 mM Tris–HCl (pH 7.5), 100 mM KCl, 1 mM DDM) added with 10 mM imidazole and 100 μM TCEP. The resin was then incubated with 10 μL of 3‐bromo‐1,1,1‐trifluoroacetone (BTFA, cat.no. 374059, Sigma) directly aliquoted from the commercially available stock at 4°C overnight, with slow shaking. The HisPur Cobalt Resin with the bound protein was washed with Buffer B added with 10 mM imidazole and LeuT was collected by elution with Buffer B containing 250 mM imidazole. LeuT was further purified by size exclusion chromatography using a Superdex 200 10/300 column.

### 2.2 ^19^F‐NMR spectroscopy

^19^F‐NMR experiments were performed using a Bruker Ascend 500 MHz spectrometer equipped with a Prodigy triple resonance cryogenic probe CRPN2—TR‐^1^H&^19^F/^13^C/^15^N‐5mm‐EZ (Bruker Instruments). Trifluoroacetic acid (50 μM) and 10% D_2_O were included in the sample and used as the chemical shift reference (−75.4 ppm) and lock reagent. 1D ^19^F‐NMR spectra were recorded with 4 k or 8 k points, a spectral width of 40 ppm and an acquisition time of 0.221 s. The carrier frequency was set to −70 ppm. The number of scans was set depending on the sample conditions, but on average was 16 k. The recycle delay was 0.6 s. A 90° pulse length was calibrated for each sample and was typically 11.5 μs. All the spectra were recorded without ^1^H decoupling. 1D spectra were processed using MestreNova 14.2.1 software (Mestrelab Research) employing a 30 Hz exponential windows function and zero filling to 16 k points. The spectra were baseline‐corrected before the fitting procedure. Peaks were fitted to generalised Lorentzian (GL) peak shapes. Fitting was performed employing the standard fitting module built in MestreNova 14.2.1 employing a 10 Hz lower width constraint and a 500 Hz upper constraint, locking shift and peak shape. Simulated annealing was employed in the fitting procedure with 10 000 maximum coarse and fine interaction. The procedure was repeated until it reached the lowest fit residual error. No sample exclusion criteria were pre‐determined, and no sample size calculations were carried out.

### 2.3 Binding assay

Leucine binding was determined for LeuT WT and K398C mutant by the ability of increasing concentrations of unlabelled Leucine to displace a fixed concentration of [^3^H]‐Leucine (homologous binding). In a clear‐bottom 96‐well plate (Corning), 0.5 μg/mL of the tested LeuT variants were mixed with 1.25 mg/mL YSi‐Cu His‐Tag SPA beads (cat.no. RPNQ0096, PerkinElmer), 100 nM [^3^H]‐Leucine (cat.no. NET1166001MC, PerkinElmer) and Assay Buffer (20 mM Tris–HCl (pH 7.5), 0.1 mM TCEP, 0.05% (w/v) DDM) supplemented with 200 mM Na^+^. Plates were sealed, mixed on an orbital shaker for 30 min at room temperature and incubated for ~16 h at 4°C. Counts per minute (c.p.m.) were recorded on a 2450 MicroBeta^2^ microplate counter (PerkinElmer). Data points were normalised to control (without competing unlabelled Leucine) and fitted to a homologous binding model (GraphPad Prism 9.0). Binding experiments were performed in triplicates.

## 3 RESULTS

### 3.1 LeuT adopts four ^19^F‐NMR‐detected distinct conformations

**FIGURE 2:**
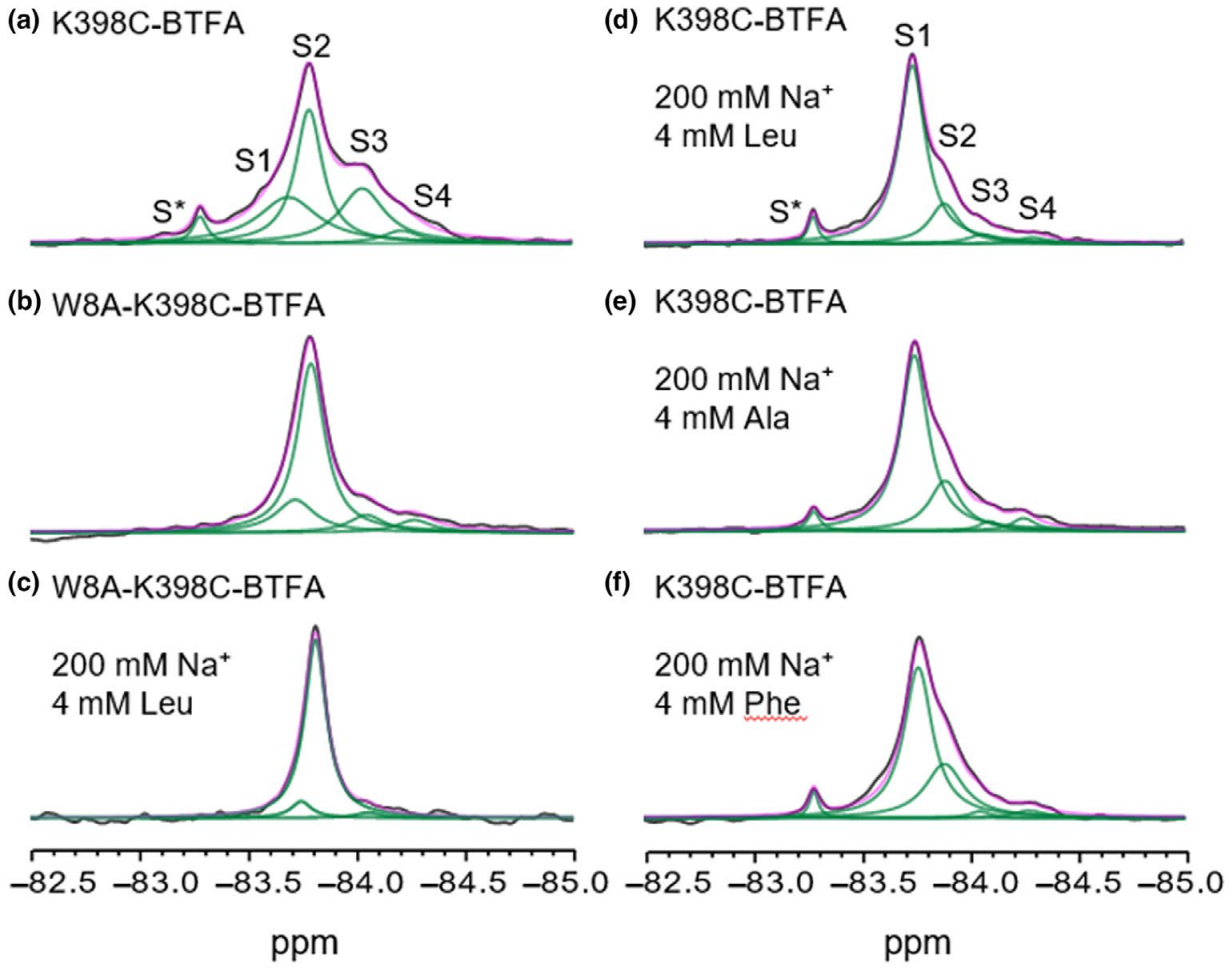
^19^F‐NMR spectra of LeuT K398C‐BTFA in detergent micelles recorded at 298 K. 1D spectra of LeuT K398C‐BTFA in the absence of Na^+^/substrates (a), LeuT W8A‐K398C‐BTFA mutant in absence of Na^+^/substrates (b), LeuT W8A‐K398C‐BTFA mutant in presence of 200 mM Na^+^ and 4 mM L‐leu (c), LeuT K398C‐BTFA in presence of 200 mM Na^+^ and 4 mM L‐leu (d), LeuT K398C‐BTFA in presence of 200 mM Na^+^ and 4 mM L‐ala (e) and LeuT K398C‐BTFA in presence of 200 mM Na^+^ and 4 mM L‐phe (f). Black line is raw NMR data, magenta is fitted data and green is deconvoluted peaks (S1–4). S* is free BTFA and it is used as a reference together with TFA peak at −75.4 (not shown in the spectra).

To enable ^19^F‐NMR studies, a fluorinated probe must be first introduced in the protein. One of the most common approaches is the functionalization of a free cysteine residue with a small probe bearing one or more fluorine nuclei (Danielson & Falke, 1996). LeuT is conveniently devoid of cysteines, so any off‐target labelling can be ruled out. We selected a LeuT mutant (K398C) that bears a cysteine residue at the end of the extracellular side of TM10 (Figure 1a) and has been shown previously to retain binding and transport activity in proteoliposome‐based assays. Moreover, previous studies based on TmFRET demonstrated that this location is a sensitive reporter of conformational rearrangements of the transporter (Figure 1b), mainly because of movements of EL4 relative to the scaffold domain in response to stimuli such as substrate/ions binding (Billesbølle et al., 2016; Schmidt et al., 2024). We confirmed that LeuT K398C binds Leucine with WT‐like affinity, as assayed by SPA assay (Figure S1). Therefore, we purified LeuT K398C in DDM detergent micelles and labelled it with BTFA (3‐bromo‐1,1,1‐trifluoropropanone) (Figure 1c) which readily reacts with the thiol group of a free cysteine residue (Chung et al., 2012; Kim et al., 2013; Luchette et al., 2002). To understand the conformational landscape of LeuT, we recorded one‐dimensional (1D) ^19^F‐NMR spectra of K398C‐BTFA LeuT (Figure 2a) in the absence of substrates and sodium ions. The spectrum of LeuT shows four main resonances with different intensities (named S1–S4) that partially overlap and do not exchange rapidly on the NMR timescale (S* is residual‐free BTFA). The relative populations of the resonances in the spectrum of LeuT K398C‐BTFA, calculated from the areas of peaks, are 29.8% (S1), 38.6% (S2), 26.6% (S3) and 5.0% (S4).

### 3.2 ^19^F‐NMR spectra distinguish outward‐/inward‐facing states

Having identified four resonances for LeuT, we set out to assign each peak in the spectrum to a specific conformational state of the transporter. Previous computational studies identified the importance of the conserved residue tryptophan 8 in closing the intracellular side of LeuT and mutation of Trp 8 to alanine (W8A) was found to shift LeuT equilibrium toward an inward‐facing (IF) conformation (Cheng & Bahar, 2014; Gotfryd et al., 2020). Therefore, we labelled W8A‐K398C‐BTFA LeuT with BTFA. The ^19^F‐NMR spectrum of W8A‐K398C‐BTFA LeuT (Figure 2b) shows a significant rearrangement of the peaks' intensities and widths. Resonance S2 is markedly increased, suggesting that this peak represents IF conformations of LeuT. All other resonances (S1, S3 and S4) have reduced intensities but do not disappear entirely from the spectrum, which indicates that the transporter is still able to explore those conformations, although the equilibrium is heavily shifted toward IF states (S2). Indeed, transport studies in proteoliposomes (Gotfryd et al., 2020) demonstrated that LeuT W8A retains the ability, albeit reduced, to transport substrates, which requires conformational exchange. Interestingly, saturating concentrations of Na^+^ in combination with the high‐affinity substrate Leucine (Leu) did not alter the conformations of the mutant (Figure 2c). To confirm our assignment of resonance S2 to the IF states of LeuT we considered mutation R5A, which abolishes the critical R5‐D369 inner salt bridge (Yamashita et al., 2005) and is known to induce an inward‐facing open (IfO) conformation of LeuT (Khan et al., 2020). We labelled LeuT R5A‐K398C with BTFA and recorded 1D ^19^F‐NMR spectra in the absence of Na^+^ (Figure S2a) and the presence of 200 mM Na^+^ (Figure S2b). The same four resonances observed for LeuT K398C are present but resonance S2 is clearly increased, an effect similar, albeit less pronounced, to the one induced by W8A mutation.

### 3.3 The diverse outward‐facing states

The earliest available structure of LeuT was crystallised with Leucine and sodium ions bound (PDB 2A65) and showed the transporter in an outward‐facing closed conformation (OfC) (Yamashita et al., 2005). Later structures of LeuT crystallised with transported substrates, such as alanine, and sodium ions also showed the transporter in an OfC conformation (Kessel et al., 2008). We therefore tested the effect that these ligands have on LeuT. The addition of saturating Leucine (Leu) to LeuT in the presence of sodium ions (200 mM Na^+^) induced a dramatic increase in resonance S1 compared to the other peaks (Figure 2d). Consequently, we assigned resonance S1 to the OfC conformation of LeuT. Our assignment is also confirmed by the ^19^F‐NMR spectrum obtained with two other ligands, Alanine/Na^+^ K398C‐BTFA LeuT (Figure 2e) and Phenylalanine/Na^+^ K398C‐BTFA LeuT (Figure 2f) which show a similar shift toward S1 resonance.

**FIGURE 3:**
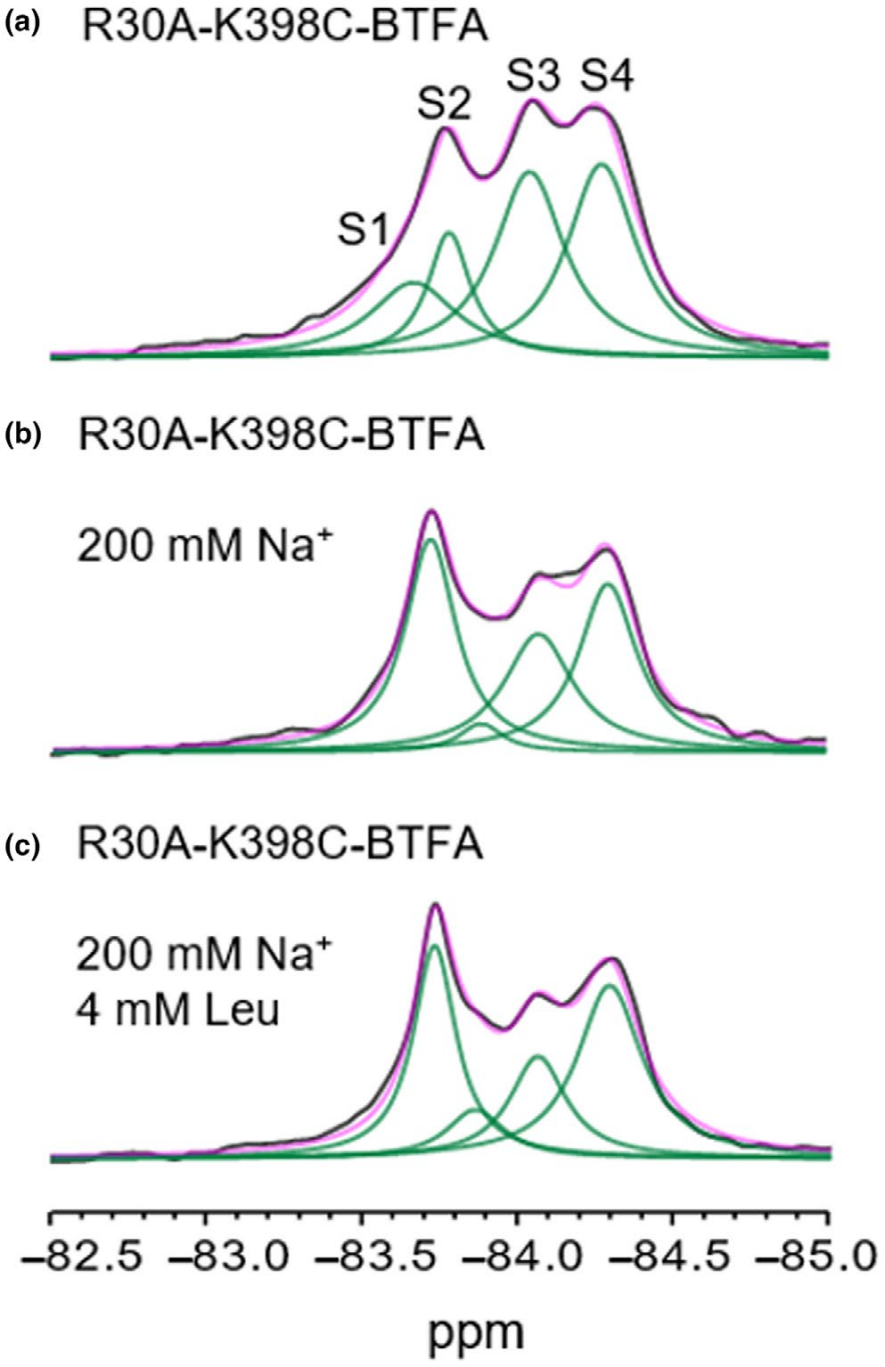
^19^F‐NMR spectra of LeuT R30A‐K398C‐BTFA reveal two diverse open states. 1D spectra of LeuT R30A‐K398C‐BTFA in the absence of Na^+^/substrates (a), in the presence of 200 mM Na^+^ (b), in the presence of 200 mM Na^+^ and 4 mM L‐leu (c). Black line indicates raw NMR data, magenta, fitted data and green, deconvoluted peaks (S1–4). All spectra were recorded at 298 K.

To assign resonances S3 and S4 to functional states we resorted to another mutation that disrupts the conformational equilibrium of LeuT. A conserved salt bridge between Arg30 (TM1) and Asp404 (TM10), located in the extracellular permeation pathway, is responsible for maintaining the substrate binding vestibule sealed during the transport cycle (Yamashita et al., 2005). Mutation of residue Arg30 has been shown to preferentially induce outward open conformations of LeuT (Billesbølle et al., 2016; Krishnamurthy & Gouaux, 2012; Zhao et al., 2010). We therefore mutated Arg30 into alanine (R30A) in the background of K398C and we labelled it with BTFA. Figure 3a shows the 1D ^19^F‐NMR spectrum of R30A‐K398C‐BTFA recorded in the absence of sodium ions and ligands. The mutant shows similar peak positions compared to K398C‐BTFA LeuT but dramatically increased intensities of resonances S3 and S4. The presence of the IF S2 and the outward‐closed S1 resonances indicates that the rupture of the salt bridge between Arg30 and Asp404 does not completely block the ability of the transporter to close the extracellular vestibule. Indeed, the addition of saturating concentrations of Na^+^ alone (Figure 3b) or in combination with Leucine (3c) induced increased S1 resonances in the R30A mutant and a general rearrangement of the peak landscape. Because R30 mutation induced both S3 and S4 resonances we cannot unambiguously assign them to a specific conformation. Several crystal structures of outward‐facing open (OfO) LeuT have been published, in combination with inhibitors and in the presence or the absence of sodium ions (Fan et al., 2021; Kessel et al., 2008; Krishnamurthy & Gouaux, 2012; Malinauskaite et al., 2016). Therefore, we posit that S3 and S4 both represent an ensemble of OfO states that are preferentially induced by the disruption of the extracellular gate (R30A mutation) but are also explored by WT LeuT (Figure 2a). The upfield chemical shift observed for S3 and S4 is consistent with greater solvent exposure of the extracellular side of TM10 (Manglik et al., 2015), which would be expected for OfO states. We also note that resonance S2, associated with IF LeuT, is greatly reduced, consistently with the notion that rupture of the outer gate selectively induces outward‐facing states at the expense of IF conformations.

### 3.4 Effect of ions on the conformational equilibrium of LeuT

**FIGURE 4:**
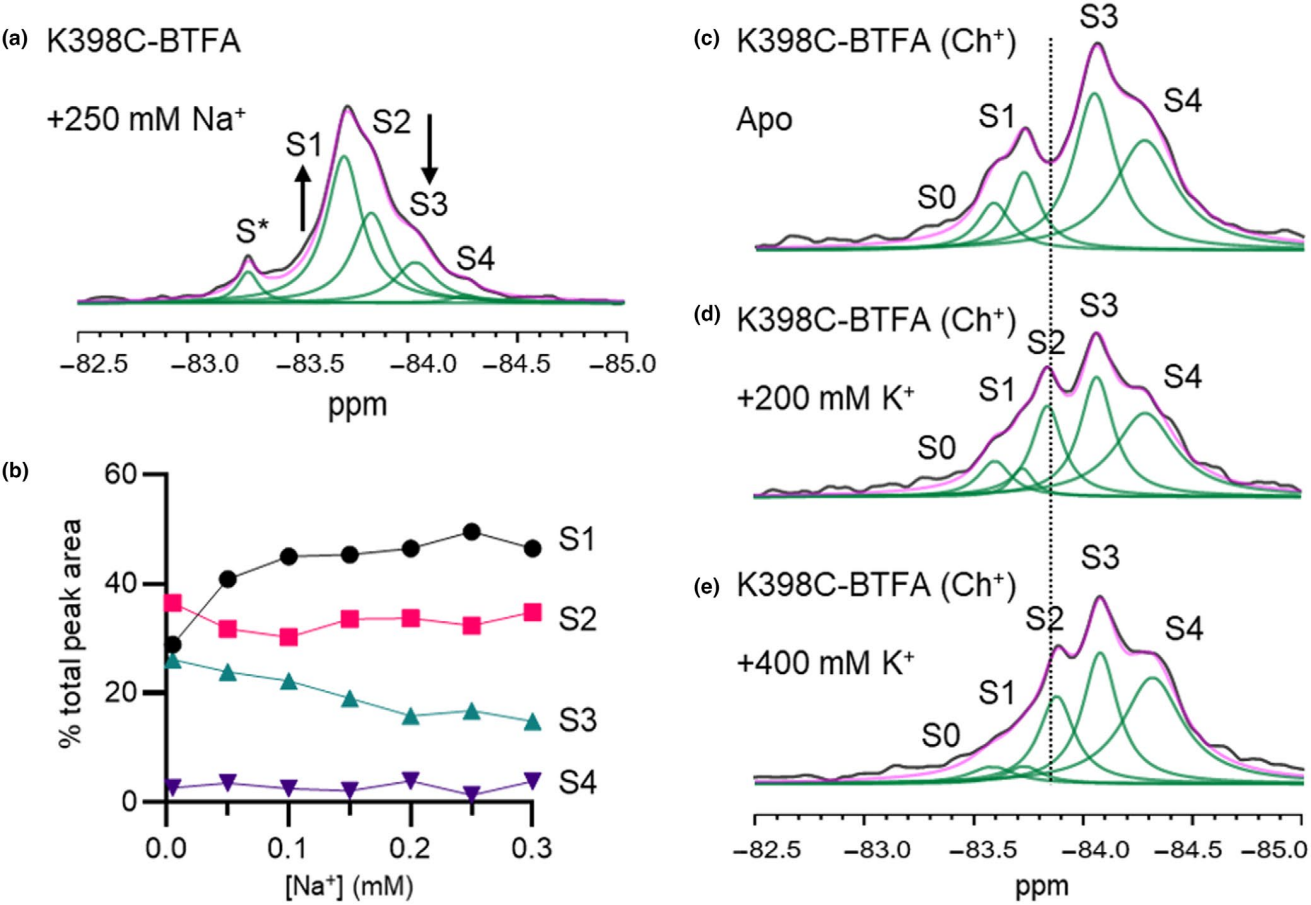
Effect of Na^+^ and K^+^ on the conformations of LeuT. (a) 1D spectra of LeuT K398C‐BTFA in the presence of 250 mM Na^+^. Black arrows highlight the effect of sodium ions on resonance S1 and S3. (b) Area of the peaks S1‐S4 measured as % of the total peak area extracted from the ^19^F‐NMR 1D spectra of LeuT K398C‐BTFA versus increasing Na^+^ concentration. (c) 1D spectra of apo LeuT K398C‐BTFA (Ch^+^ sample) purified in the presence of 100 mM choline. (d) 1D spectra of LeuT K398C‐BTFA (Ch^+^ sample) in presence of 200 mM K^+^. (e) 1D spectra of LeuT K398C‐BTFA (Ch^+^ sample) in presence of 400 mM K^+^. Black line is raw NMR data, magenta is fitted data and green is deconvoluted peaks (S0–4). All spectra were recorded at 298 K (e).

Prompted by our assignment of the resonances in the spectrum of LeuT K398C‐BTFA and to clarify the roles of the different conformations in LeuT mediated transport we investigated the effect that Na^+^ ions alone have on the resonances of LeuT K398C‐BTFA. Figure 4a shows the 1D ^19^F‐NMR spectrum after the addition of saturating concentrations of Na^+^. The binding of sodium ions visibly affected the resonances of the transporter, seemingly inducing the same OfC S1 conformation stimulated by the transported substrates alanine and leucine in the presence of sodium. Because the difference in chemical shift (Δδ) between S1 and S2 is relatively small (~0.1 ppm) and the addition of large amounts of salts can non‐specifically affect the position of the resonances we titrated increasing amounts of Na^+^ ions on LeuT K398C‐BTFA and followed its effect on the conformations, monitoring the area of the four peaks (Figure 4b; Figure S3a). We found that sodium ions mostly affect the transition from the OfO state to the OfC state. Interestingly, we observed only a small reduction of resonance S2 (IFs).

Studies based on TmFRET (Billesbølle et al., 2016; Schmidt et al., 2024) have suggested a role for potassium ions in regulating the conformational equilibrium of LeuT, inducing closure of the extracellular gate and competing with Na^+^ for binding at the Na1 site. Because K^+^ has a lower affinity for LeuT compared to Na^+^ and they compete for the same site (Billesbølle et al., 2016; Schmidt et al., 2024), it would be arduous to observe potassium‐mediated conformational changes in the presence of Na^+^. We therefore prepared samples of LeuT purified and labelled in the presence of the counterion choline chloride (Ch^+^) which maintains the ionic strength and does not interact with LeuT (Billesbølle et al., 2016; Schmidt et al., 2024). This allowed us to obtain the 1D ^19^F‐NMR spectrum of the ‘true’ apo LeuT (Figure 4c). In the nominal absence of any interacting ligands/ions, we observed three peaks, which we assigned to the already identified S1 (OfC) and S3/S4 (OfO) resonances, plus a new additional peak, named S0. The addition of K^+^ at concentrations close to the estimated EC50 (200 mM, Figure 4d) and twice its value (400 mM, Figure 4e) induced the appearance of resonance S2 (IFs) and decreased the intensity of resonances S1 (OfC) and S0 (not assigned) while leaving resonances S3/S4 (OfO) unaffected. This is direct evidence that the potassium ions not only bind to LeuT but also affect the conformational equilibrium of LeuT shifting it toward IF intermediates (i.e. S2).

## 4 DISCUSSION

To understand transmembrane transport it is fundamental to investigate the dynamic conformational states underlying its mechanism. ^19^F‐NMR is a simple, sensitive technique perfectly suited for such a task. Recently, ^19^F‐NMR has been applied to distinguish conformational ensembles, extrapolate exchange dynamics and obtain distance information in membrane proteins (Huang et al., 2020; Manglik et al., 2015; Matei & Gronenborn, 2016). In this study, we applied ^19^F‐NMR to investigate the effect of substrates/ions on the conformation landscape of DDM solubilised LeuT. While it should be noted that detergent micelles are not the natural transporter environment, we chose to use detergent solubilised LeuT to adhere to the previous rich literature on the function and structure of LeuT and LeuT‐fold transporters. To study LeuT with ^19^F‐NMR we introduced a fluorine nucleus at a location (K398) highly sensitive to the movements of EL4, that is transitions from IfC/IfO → OfO → OfC (Figure 1a,b). Moreover, to make sure that the observed resonance changes could be credited to specific LeuT conformations we employed conditions (e.g. ion/substrates) that are known to produce large conformational changes in the vicinity of EL4.

1D ^19^F‐NMR of LeuT K398C‐BTFA showed four distinct resonances that do not undergo rapid exchange (Figure 2a). The appearance of several peaks and, thus, the fact that LeuT explores more than one conformation is not surprising. Previous smFRET and DEER studies have demonstrated that LeuT can undergo slow spontaneous conformational exchanges in detergent micelles and the nominal absence of sodium ions and substrates (Kazmier et al., 2014; Terry et al., 2018). By introducing mutations (W8A and R5A) at the cytoplasmic side of LeuT that are known to favour IF intermediates we assigned peak S2 to the IfO and inward facing closed (IfC) states (Figure 2b,c; Figure S2a,b). The fact that a single sharp peak can be assigned to both IfC and IfO states of the transporter is not unexpected. Close inspection of the crystal structure of the IfC (PDB 6XWM) and IfO (PDB 3TT3) structures of LeuT reveals that the extracellular end of TM10 is not directly involved in the structural rearrangement responsible for the opening‐closing of the intracellular gate (Gotfryd et al., 2020) and it is barely affected by such movements. Therefore, the difference in the chemical environment surrounding the fluorinated probe in the IFO and IFC state is likely not diverse enough for us to observe a detectable chemical shift change associated with the opening of the intracellular gate and thus explain why we observed a single peak for the inward facing conformations (Ifs). On the extracellular side of the transporter, disruption of the interaction between Arg30 and Asp404 has been associated with the outward opening of LeuT (Billesbølle et al., 2016; Khan et al., 2020; Yamashita et al., 2005). Mutation of Arg30 into alanine induced a reduction of peak S2, consistent with our assignment of this resonance to the IF intermediates, as well as a marked increase in resonance S3 and S4 (Figure 3a). Several crystal structures of LeuT in the OfO state have been published, in the presence of inhibitors and with/without sodium bound. While they all share a high degree of similarity, they also display different levels of ‘openness’ of the extracellular vestibule. The location of our ^19^F reporter probe makes it extremely sensitive to structural rearrangements happening at the extracellular side of TM10. Therefore resonance S3 and S4 probably represent two different OfO states of LeuT. At the moment we cannot uniquely identify these resonances but both peaks are also present in apo LeuT K398C‐BTFA indicating that they are indeed functional states explored by the wild‐type transporter. With this assignment of the resonances, we tested the effect of the important sodium ion on the conformations of LeuT (Figure 4a). Titrating Na^+^ on apo LeuT K398C‐BTFA and monitoring the area of the peaks in the 1D ^19^F NMR spectra (Figure 4b) revealed that higher concentrations of Na^+^ induced an increase of S1 and a reduction of resonance S3, a similar effect generated by the presence of Na^+^ and substrates (Figure 2d–f). The fact that Na^+^ alone and Na^+^/substrates seem to induce the same OfC state (Figures 2d–f and 4a) is somewhat surprising. Studies based on EPR (Claxton et al., 2010; Kazmier et al., 2014) and cysteine accessibility (Tavoulari et al., 2016; Zhang et al., 2018) observed looser packing and opening of the extracellular vestibule of LeuT following Na^+^ binding. Computational studies (Zhao et al., 2012) also identified a role for sodium ions in inducing the outward opening of the extracellular vestibule. Nonetheless, in recent smFRET studies (Terry et al., 2018) Na^+^ binding in apo LeuT was found to induce a compaction of the extracellular vestibule and reduced the lifetimes of the OfO state(s). Our NMR data (Figure 4a,b) agree with those results and argue that (i) Na^+^ binding to LeuT induces an outward‐facing state, that (ii) this state is closely related to the conformation induced by the concomitant presence of Na^+^/ligands and finally that (iii) this state is considerably diverse from the OfO states induced by disruption of the extracellular gate (i.e. R30A mutant). Following recent evidence of a role for potassium ions in regulating the transport mechanism of LeuT (Billesbølle et al., 2016; Schmidt et al., 2024) we evaluated its effect on the ^19^F‐NMR resonances. To measure the effect of K^+^ we prepared a ‘true’ apo‐LeuT sample without any interacting ions/substrates (Ch^+^ sample). Interestingly, under these conditions, LeuT equilibrium is almost completely shifted toward S1 and S3/S4 resonances assigned to OfC and OfOs states respectively (Figure 4c). This argues that in the absence of Na^+^/K^+^ and ligands, LeuT preferentially adopts outward‐facing states. We also detected a new small peak, termed S0, but we were unable to assign it to any specific state of LeuT. The addition of K^+^ to apo LeuT caused the disappearance of peak S1 (and S0) and the concomitant appearance of peak S2 (Figure 4d,e). As demonstrated by the effect on the fluorine resonance of LeuT, potassium ions clearly interact with the transporter and induce a shift in the conformational equilibrium toward IF intermediates as seen by the increased S2 resonance (Figure 4c–e). It could be argued that the changes in peak intensities in Figure 4d,e might be induced by K^+^ binding to the If state and the result of K^+^‐bound and K^+^‐free states, with both states observable because of slow chemical exchange in the NMR timescale. Nonetheless, because of the higher affinity of Na^+^ for LeuT compared to K^+^, the chemical exchange between Na^+^‐bound and Na^+^‐free states should be slower than for K^+^ and the effect should be even more evident for Na^+^. This is not the case, as can be observed in Figure S3. Thus, our NMR results reinforce the hypothesis (Billesbølle et al., 2016; Schmidt et al., 2024) that K^+^ can affect the transport cycle of LeuT by (i) competing for binding at the sodium binding site Na1 and (ii) inducing IF conformations.

Traditionally, the application of liquid‐state NMR to the investigation of large membrane transporters has been comparatively limited because of signal broadening and poor signal‐to‐noise ratios. Alternative approaches, such as ^19^F‐NMR or STD‐NMR (Mayer & Meyer, 1999), have emerged. NMR investigation of fluorine resonances is an extremely promising avenue not only because of its inherent physical properties but also because fluorine incorporation into protein structures is generally non‐perturbing (Danielson & Falke, 1996). This is because of the small volume of the fluorine nuclei and fluorinated probes (Gronenborn, 2022), allowing its positioning in critical locations of the protein structure without compromising its activity. We note that, while this approach is able to afford meaningful data on transient conformational states and their population changes, it requires pre‐existing structural data to complement the peak assignment. In our case, we began the deconvolution process with four resonances which is the minimum meaningful number that corresponded to the expected number of LeuT ground states (IfO, IfC, OfC and OfC). Because these peaks consistently appeared in the subsequent LeuT experiments with mutations, substrates and ions that affected LeuT states, we could confirm our assignment of four main resonances. Nonetheless, we are well aware that additional peaks might exist and that we could not identify them because of their low population and/or fast exchange or simply because they belong to conformations not explored by the transporter under our experimental conditions.

In the present work, we applied the NMR technique based on the observation of ^19^F‐NMR resonances to the membrane transporter protein LeuT. This is the first instance where NMR is being used to investigate the conformational properties in response to ion/substrate interactions of a LeuT‐fold member. Human transporters belonging to this structural fold (e.g. DAT, SERT, NET, GAT, etc.) have been implicated in a variety of debilitating pathophysiological conditions. Given the unique ability of NMR to elucidate relevant but transient conformational states, we trust that the addition of NMR into the toolkit of biophysical techniques employed to investigate LeuT‐fold transporters will benefit the current knowledge on these proteins.

## AUTHOR CONTRIBUTIONS

**Alberto Daminato:** Writing – original draft; writing – review and editing; methodology; formal analysis; conceptualization; investigation; validation. **Claus J. Loland:** Funding acquisition; writing – review and editing; supervision. **Eurico J. Cabrita:** Funding acquisition; supervision; conceptualization; writing – review and editing; project administration; investigation; formal analysis; validation.

## FUNDING INFORMATION

This work was supported by European Union's Horizon 2020 research and innovation programme under the Marie Skłodowska‐Curie grant agreement No: 860954 and by Fundação para a Ciência e a Tecnologia (FCT‐Portugal) UCIBIO project (UIDP/04378/2020 and UIDB/04378/2020) and Associate Laboratory Institute for Health and Bioeconomy—i4HB project (LA/P/0140/2020). The NMR spectrometers are part of the National NMR Facility supported by FCT‐Portugal (ROTEIRO/0031/2013–PINFRA/22161/2016, co‐financed by FEDER through COMPETE 2020, POCI and PORL and FCT through PIDDAC).

## CONFLICT OF INTEREST STATEMENT

The authors declare no conflicts of interest.

### PEER REVIEW

The peer review history for this article is available at https://www.webofscience.com/api/gateway/wos/peer‐review/10.1111/jnc.16278.

## Supporting information


Appendix S1.

## Data Availability

The data that support the findings of this study are available from the corresponding author upon reasonable request.
