# Supplementary material for: Monitoring conformational changes in the human neurotransmitter transporter homologue LeuT with 19F‐NMR spectroscopy
Source: J Neurochem. 2024 Dec 16;169(1):e16278. doi: 10.1111/jnc.16278 (PMC11649037; doi:10.1111/jnc.16278)
Supplement: Supplementary file 1 — Appendix S1. [file JNC-169-0-s001.docx]

**Monitoring conformational changes in the human neurotransmitter transporter homolog LeuT with ^19^F-NMR spectroscopy**

Alberto Daminato^1,2^, Claus J. Loland^3^,  Eurico J.Cabrita^1,2*^

^1^Associate Laboratory i4HB - Institute for Health and Bioeconomy, NOVA School of Science and Technology, Universidade NOVA de Lisboa, 2829-516 Caparica, Portugal;

^2^UCIBIO, Department of Chemistry, NOVA School of Science and Technology, Universidade NOVA de Lisboa, 2829-516 Caparica, Portugal;

^3^Laboratory for Membrane Protein Dynamics, Department of Neuroscience, Faculty of Health and Medical Sciences, University of Copenhagen, Copenhagen, Denmark

***Corresponding author:** Eurico J. Cabrita

**Supplementary Material**


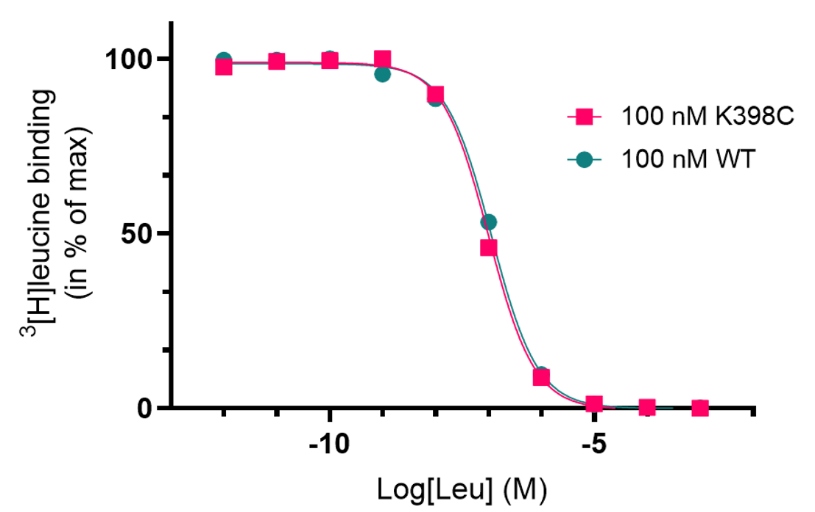


**Supplementary figure S1. LeuT WT and K398C have similar Leucine binding affinity.**

Displacement of [^3^H]Leucine (100 nM) binding by Leucine for LeuT WT (green circles) and K398C (magenta squares). Binding was assayed in 200 mM Na^+^. Error bars smaller than data points. All data points are shown as mean ± standard error of the mean (s.e.m.), conducted in triplicates.


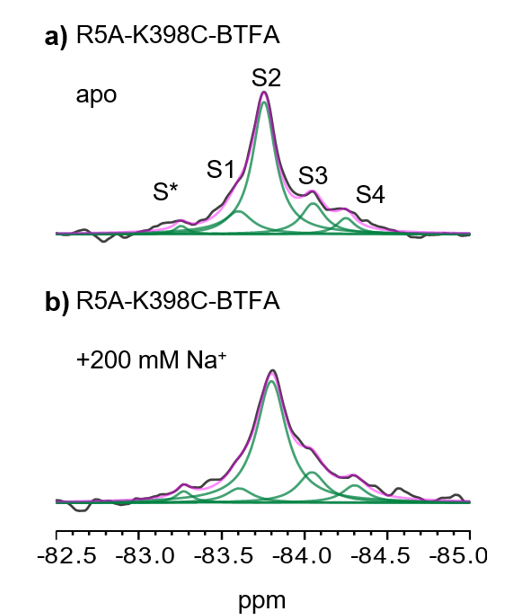


**Supplementary figure S2. ^19^F-NMR spectra of LeuT R5A-K398C-BTFA in detergent micelles recorded at 298 K.** 1D spectra of LeuT R5A-K398C-BTFA in the absence of Na^+^/substrates (**a**), and in presence of 200 mM Na^+^ (**b**). Black line is raw NMR data, magenta is fitted data and green is deconvoluted peaks (S1-4). S* is free BTFA and it is used as reference together with TFA peak at -75.4 (not shown in the spectra).


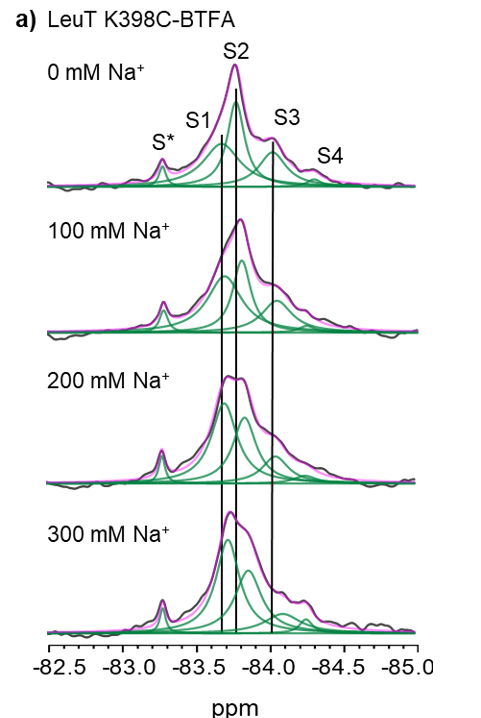
We noted that Na^+^ produced a rightward movement of the chemical shift of the LeuT resonances in a concentration dependent manner. We propose that this is an unspecific effect due to the local increase in ionic strength surrounding the fluorinated probe following interaction of Na^+^ with the transporter/micelle system. This is best observed when considering some of the titration steps that have been used to generate figure 4b in the manuscript (supplementary figure S3, see below). With the aid of the vertical black lines positioned at S1, S2 and S3 it is possible to appreciate how increasing Na^+^ produces an upfield shift of the resonances. In figure 2 of the manuscript all the 1D ^19^F NMR spectra on the right column were collected in presence of 200 mM Na^+^ which explain the rightward shift of the resonances.

**Supplementary figure S3. ^19^F-NMR spectra of LeuT K398C-BTFA in detergent micelles recorded at increasing NaCl concentrations. (a)** 1D spectra of LeuT K398C-BTFA in the absence of Na^+^/substrates (top), in presence of 100 mM Na^+^ (middle-top), 200 mM Na^+^ (middle-bottom), and 300 mM Na^+^ (bottom).
